# Improving targeted small molecule drugs to overcome chemotherapy resistance

**DOI:** 10.1002/cnr2.1945

**Published:** 2023-11-22

**Authors:** Amirhossein Rismanbaf

**Affiliations:** ^1^ Department of Cellular and Molecular Biology, Faculty of Advanced Science and Technology, Tehran Medical Sciences Islamic Azad University Tehran Iran

**Keywords:** cancer stem cell, chemotherapy resistance, DNA repair mechanisms, immunomodulation, small molecule, tumor microenvironment

## Abstract

**Background:**

Conventional cancer treatments face the challenge of therapeutic resistance, which causes poor treatment outcomes. The use of combination therapies can improve treatment results in patients and is one of the solutions to overcome this challenge. Chemotherapy is one of the conventional treatments that, due to the non‐targeted and lack of specificity in targeting cancer cells, can cause serious complications in the short and long‐term for patients by damaging healthy cells. Also, the employment of a wide range of strategies for chemotherapy resistance by cancer cells, metastasis, and cancer recurrence create serious problems to achieve the desired results of chemotherapy. Accordingly, targeted therapies can be used as a combination treatment with chemotherapy to both cause less damage to healthy cells, which as a result, they reduce the side effects of chemotherapy, and by targeting the factors that cause therapeutic challenges, can improve the results of chemotherapy in patients.

**Recent Findings:**

Small molecules are one of the main targeted therapies that can be used for diverse targets in cancer treatment due to their penetration ability and characteristics. However, small molecules in cancer treatment are facing obstacles that a better understanding of cancer biology, as well as the mechanisms and factors involved in chemotherapy resistance, can lead to the improvement of this type of major targeted therapy.

**Conclusion:**

In this review article, at first, the challenges that lead to not achieving the desired results in chemotherapy and how cancer cells can be resistant to chemotherapy are examined, and at the end, research areas are suggested that more focusing on them, can lead to the improvement of the results of using targeted small molecules as an adjunctive treatment for chemotherapy in the conditions of chemotherapy resistance and metastasis of cancer cells.

## 1 INTRODUCTION

**FIGURE 1:**
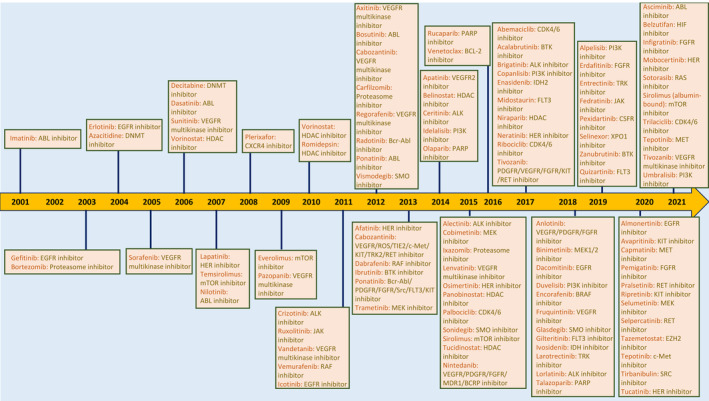
Approved targeted small molecule drugs by the FDA for cancer treatment from 2001 to 2021.

Cancer is a heterogeneous and highly complex disease that annually claims the lives of millions of people, making it one of the leading causes of death worldwide.^1^ The number of deaths caused by cancer is growing increasingly and predictions indicate that it will continue to do so. Estimates show that in 2018 and 2021, 9.6 million and more than 10 million cancer‐related deaths occurred respectively.^2^ However, by 2030, it is projected that around 30 million people may die annually from cancer.^2^ That is why cancer is a very serious challenge for public health worldwide, and it becomes increasingly widespread and severe every year. Although there are innovative and diverse methods for cancer treatment, four treatment methods are conventional and usually employed to combat cancer, including surgery, radiation therapy, immunotherapy, and chemotherapy. Chemotherapy, however, faces many challenges such as non‐specific targeting of cancer cells, treatment resistance, and cancer recurrence even after successful treatment. The use of targeted small‐molecule drugs is one of the ways to improve the outcome of chemotherapy. Because of their low molecular weight (<1000 Da) and small size, they can bind to various targets outside and inside the cell.^3^ Since 2001, when the first small molecule tyrosine kinase inhibitor (TKI) drug, imatinib, by the US Food and Drug Administration (FDA), was approved for clinical use, more than 80 small molecule drugs for cancer treatment have been approved by the US FDA and the National Medical Products Administration (NMPA) of China^4^ Figure 1. However, the therapeutic results of targeted small molecules are limited. Cancer cells use a variety of factors and strategies to fight and resist chemotherapy. For this reason, to improve the results of treatments based on small molecules as adjunctive therapy to overcome the challenges of chemotherapy, knowledge and deep understanding of chemotherapy resistance mechanisms and then determining the most effective and main factors as targets for small molecules is very crucial and important. In this study, most of these factors and mechanisms are comprehensively examined in four titles including tumor microenvironment (TME), immunomodulation, DNA repair mechanisms, and cancer stem cells (CSCs). It is crucial and essential for drug designers and scientists to extensively investigate these mechanisms for the proper and effective design of targeted small molecule drugs as adjunctive chemotherapy treatments.

## 2 TUMOR MICROENVIRONMENT

The TME provides a secure environment for cancer cells to evade the desired outcomes of various treatments. Essentially, the TME is a complex and dynamic ecosystem composed of diverse factors that play crucial roles in inhibiting apoptosis, proliferation, migration, immune evasion, treatment resistance, metastasis, metabolic reprogramming, and all stages of tumorigenesis.^5^ The TME factors are generally divided into two main components: cellular components (such as tumor‐associated macrophages [TAMs], tumor‐infiltrating lymphocytes [TILs], myeloid‐derived suppressor cells [MDSCs], cancer‐associated fibroblasts [CAFs], and endothelial cells [ECs] which all of them are stromal cells), and non‐cellular components (such as growth factors, various chemokines and cytokines, interstitial fluids, metabolites, extracellular matrix [ECM] and exosomes).^5^, ^6^ Therefore, targeting the TME is a potentially effective strategy for achieving fruitful outcomes of cancer therapy, and small molecules can easily penetrate the TME and ultimately reach tumor cells and affect them.^6^ The TME is a hypoxic and low‐pH environment.^6^ The rapid growth of tumor cells causes hypoxia, which subsequently causes the release of stimulating factors such as vascular endothelial growth factor, matrix metalloproteinases (MMPs), and hypoxia‐inducible factor‐1α (HIF‐1α).^5^ Also, the condition of hypoxia in TME affects the ECs by secreting angiogenic factors from the tumor and subsequently promoting angiogenesis.^7^, ^8^ In the TME, the most abundant stromal cells are CAFs that can produce abnormal ECM which supports tumor cell migration.^9^ TME reshaping creates the conditions for tumor cells to interact with surrounding fibroblasts, immune cells, and ECs, leading to the induction of a variety of biological events, including angiogenesis, migration, proliferation, immune system suppression, and drug resistance, which ultimately causes tumor growth.^9^, ^10^, ^11^, ^12^, ^13^, ^14^, ^15^, ^16^, ^17^, ^18^

One of the important events in the TME is cell interaction and cell communication with the ECM because this interaction causes the release of factors that play a role in ECM remodeling and immune evasion, which ultimately strengthens treatment resistance.^19^ Other important events are the generation of exosomes by benign and malignant cells, TME‐specific metabolic patterns, and circulating deregulated microRNAs that increase treatment resistance.^19^ There are many different types of immune cells in the TME that block the immune response, in addition, around the tumor cells, there are a set of inflammatory molecules that cause the failure to identify and eliminate cancer cells by the immune system, which together make TME a complex and heterogeneous space and they often cause an uncontrollable process in the growth and development of tumors.^5^, ^20^, ^21^, ^22^, ^23^, ^24^ On the whole, a wide range of events and factors from biochemical agents, and a hypoxic environment to abnormal mechanical forces cause treatment resistance.^19^ In the following, circumstances and various components of the TME are discussed.

### 2.1 Extracellular vesicles

Extracellular vesicles (EVs) act as intermediaries in intercellular communication and are secreted by various types of cells.^25^ These vesicles are composed of a lipid bilayer that protects their contents from enzymatic degradation.^26^ They carry diverse biological active molecules such as lipids, proteins, and various nucleic acids including microRNA (miRNA) and long non‐coding RNA (lncRNA), and can regulate cellular processes and functions, leading to changes in gene expression and activation of multiple signaling pathways.^25^, ^27^, ^28^, ^29^ Tumor‐derived EVs can modulate the TME.^30^ EVs by transferring surface markers and signaling molecules, nucleic acids, and oncogenic proteins to stromal cells, can prepare the TME for tumor growth, invasion, and metastasis.^31^, ^32^, ^33^ Stromal and tumor cells, by releasing EVs, cause heterogeneity and complexity in the TME.^25^ EVs create favorable conditions in the TME for tumor growth and resistance to anti‐cancer drugs by transferring bioactive materials.^25^ EVs contribute to drug resistance through various mechanisms, including drug export and sequestration, reduction of drug concentration in target sites, transferring drug efflux pumps, mediating communication between cancer and stromal cells, transfer of survival factors, apoptosis inhibitors, and non‐coding RNAs.^25^ EVs not only play a role in metastasis and drug resistance but also in immune suppression and angiogenesis, and also, by providing growth factors such as transforming growth factor‐β (TGF‐β) and various miRNAs, they can convert mesenchymal stem cells (MSCs) and other bone marrow‐derived cells into tumor‐supporting cells.^25^, ^34^, ^35^

One class of EVs is exosomes, which are involved in various cancer events such as apoptosis escape, immune suppression/evasion, cell proliferation, inflammatory responses, angiogenesis, invasion, metastasis, and chemotherapeutic sensitivity.^5^, ^36^, ^37^, ^38^, ^39^ Not only are exosomes involved in acquired drug resistance through various cellular and molecular processes in the TME (including DNA repair, epithelial‐mesenchymal transition [EMT], immune surveillance, and cell cycle), but also they contribute to drug resistance through various pathways, including transporting drug efflux pumps, direct drug export, and miRNA signaling.^40^, ^41^, ^42^ By transferring ABC transporters (drug efflux pumps) through exosomes, drug resistance is promoted in sensitive cells.^36^

#### 2.1.1 Exosomal miRNAs

**FIGURE 2:**
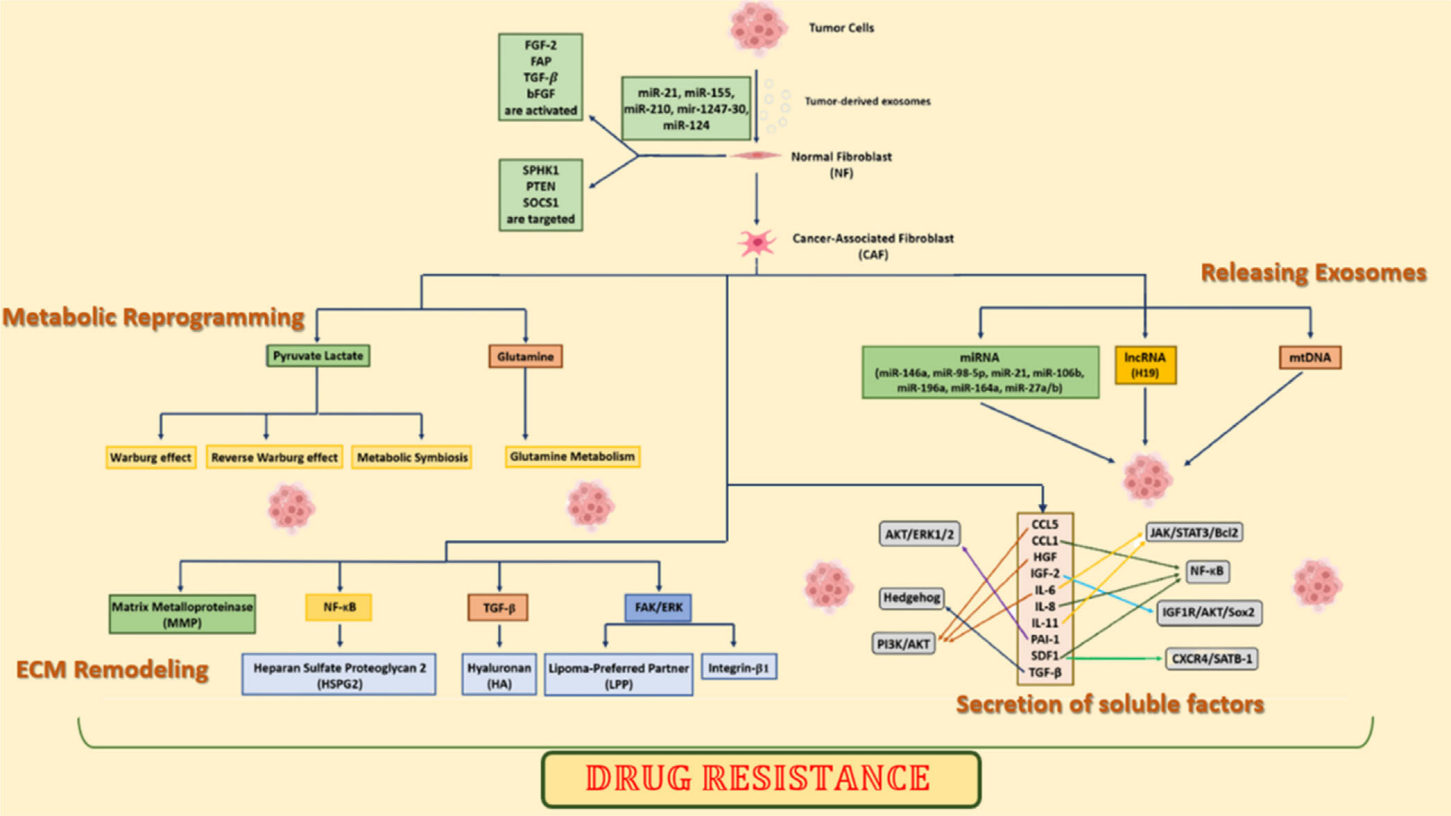
Normal fibroblasts (NFs) conversion into cancer‐associated fibroblasts (CAFs) and the role of CAFs in drug resistance.

miRNAs, or microRNAs, are a type of short non‐coding RNAs (ncRNAs) that regulate various important biological functions such as apoptosis, migration, proliferation, differentiation, drug resistance, and invasion by regulating gene expression.^5^, ^43^ Cancer cells create an abnormal expression of miRNAs through genetic or epigenetic changes, which subsequently leads to abnormal expression of their target genes.^44^, ^45^, ^46^, ^47^, ^48^, ^49^ miRNAs act as elements that promote the formation and biological changes of TME.^50^, ^51^, ^52^, ^53^ Exosomal miRNAs derived from tumors cause heterogeneity and phenotypic changes in TME and subsequently promote uncontrolled tumor growth.^20^, ^36^, ^54^, ^55^, ^56^, ^57^, ^58^ Exosomal miRNAs derived from tumors by matrix reprogramming in TME create a context for resistance to chemotherapy, tumor growth, immune system escape, and metastasis.^5^ Exosomal miRNAs secreted by CSCs that target immunosuppressive and anti‐apoptotic pathways can impart and develop drug resistance in susceptible neighboring cells.^36^ Exosomal miRNAs derived from CSCs cause inhibition of pro‐apoptotic FOXO3a property and activation of mTOR signaling pathway in sensitive cancer cells and they can inhibit apoptosis and subsequently promote tumor progression, therefore, drug‐induced apoptosis is inhibited.^36^ Horizontal transfer of exosomal miRNAs derived from cancer cells can induce a resistant phenotype of drug‐resistant cells in sensitive cancer cells and create resistance to a wide range of anticancer drugs, moreover, miRNAs can be delivered from cancer cells to TME cells by exosomes and modulate the process of drug resistance response in TME.^59^, ^60^, ^61^ Exosomal miRNAs derived from CSCs and non‐cancerous cells help to drug resistance by creating different effects on target cells in TME, in addition, exosomal miRNAs play a role in inducing resistance to specific molecular target drugs and cytotoxic drugs.^36^ Due to the key role of miRNAs in cancer and their regulation of drug resistance in a tumor‐specific manner by some miRNAs, exosomal miRNAs can be considered and used as potential cancer biomarkers for prediction and diagnosis in a broad or specific tumor approach.^36^ Exosomal miRNAs derived from cancer cells and transferred to fibroblasts in the TME promote differentiation of CAFs, subsequently, exosomal miRNAs secreted by CAFs induce drug resistance in cancer cells through induction of proliferation, metastasis, and inhibition of anti‐tumor effects of cytotoxic drugs such as cell cycle arrest and apoptosis.^36^ For example, the transfer of exosomal miR‐21 derived from CAFs to ovarian cancer cells led to inhibition of apoptosis and downregulation in the expression of apoptotic peptidase activating factor 1 (APAF1), as a result, resistance to treatment with paclitaxel was increased.^62^ Alterations in the ECM promote the widespread growth of cancer cells, angiogenesis, metabolic reprogramming, and inflammatory response.^5^ Primary tumor cells release exosomal miRNAs such as miR‐21, miR‐155, miR‐210, miR‐1247‐30, and miR‐124 that are transferred to normal fibroblasts (NFs), then, by targeting proteins such as SPHK1, PTEN, and SOCS1, as well as activating molecules such as FGF‐2, FAP, TGF‐β, and bFGF induce NFs conversion into CAFs, ultimately, ECM undergoes reshaping^5^, ^36^ Figure 2.

##### Exosomal miRNAs and tumor‐associated macrophages

The most abundant immune cell population in the TME is TAMs.^36^ TAMs are highly plastic cells that are involved in various actions, including suppressing the immune system, promoting tumor angiogenesis, and increasing resistance of tumor cells to chemotherapy.^63^, ^64^ Poor prognosis in many cancer types is directly related to the number of TAMs in the TME.^65^ According to several studies, in several cancer types, including lung, skin, colon, head and neck, bladder, and ovarian cancer, exosomal miRNAs secreted by cancer cells can induce the transition of macrophages to the TAMs.^66^, ^67^, ^68^, ^69^, ^70^, ^71^

##### Exosomal miRNAs and epithelial‐mesenchymal transition

Epithelial‐mesenchymal transition is a process in which cancer cells acquire increased motility and tremendous plasticity, caused by loss of cell junctions and apical‐basal polarity and acquisition of mesenchymal characteristics.^72^, ^73^ EMT cells are directly related to metastasis, cancer progression, and drug resistance in various cancers because they have an extremely invasive phenotype.^74^ There is ample evidence to demonstrate that the EMT process in the TME can be modulated by exosomes released from cancer cells.^41^, ^75^, ^76^ Xiao et al. showed that exosomal miRNAs involved in regulating EMT, such as miR‐191 and let‐7a derived from melanoma, induced EMT in primary melanocytes.^77^ Additionally, studies have shown that exosomal miRNAs are involved in activating or stabilizing EMT in primary tumor cells.^36^

##### Exosomal miRNAs and autophagy

Autophagy is a catabolic process that removes damaged or redundant macromolecules and organelles to maintain homeostasis and metabolic adequacy.^78^ This process is involved in escalating tumor resistance against therapy and increasing tumor growth, as well as upregulating autophagy frequently occurring leading to cell survival and high energy supply during cancer initiation.^36^, ^79^, ^80^ During a phenomenon called cytoprotective autophagy a process caused by high levels of autophagy and the creation of hypoxic TME, resulting in oxidative stress, ultimately delays apoptosis and subsequently contributes to treatment resistance.^81^, ^82^ Upregulation of exosomes and autophagy can act to induce an adaptive response under cellular stress conditions in cancer cells.^36^ Exosomes released from cancer cells can induce the construction of reactive oxygen species (ROS) and stimulate the secretion of tumor growth factors by upregulation of autophagy in target cells.^83^ Moreover, during a drug treatment regimen, exosomes derived from cancer cells cause a resistant phenotype in target cells by upregulation of cytoprotective autophagy.^84^ Exosomal miRNAs can control autophagy and play a role as mediators in therapeutic resistance.^36^, ^85^, ^86^ For example, cisplatin‐resistant non‐small cell lung cancer (NSCLC) cells secreted exosomal miR‐425‐3p, which targeted the AKT1/mTOR signaling pathway and subsequently led to the upregulated autophagy activity and reduced cisplatin treatment outcomes.^87^

#### 2.1.2 Exosomal lncRNAs (long non‐coding RNAs)

The transfer of exosomal lncRNAs between the TME and tumor cells is involved in events such as reprogramming the TME, growth, migration, and survival of cancer cells, as well as the development of mechanisms that cause resistance to chemotherapy.^88^ In addition, because exosomal lncRNAs play a role in malignancy and response to treatment, they could be employed as biomarkers for many cancers.^88^ There is evidence that lncRNAs promote drug resistance in diverse cancers.^88^ For instance, lncRNA SBF2 (SET binding factor 2) induces temozolomide resistance in glioblastoma cells during chemotherapy.^89^ During tumor growth, access to oxygen is limited due to poor vasculature within the developing solid tumor mass, which is called hypoxia.^88^ Under hypoxia conditions, the hypoxia‐inducible factor (HIF)‐1α pathway is activated in tumor cells, which during oxygen stress, plays a role as an adaptive mechanism.^90^ Moreover, the hypoxia condition promotes cell survival through the transcription of several lncRNAs.^91^, ^92^

##### Exosomal lncRNAs and autophagy

Because of the replenishment of energy supply and protection from environmental stress, cancer cells upregulate autophagy, accordingly, autophagy is a significant event in cancer.^93^ Studies have shown that because lncRNAs are key regulators of autophagy, they can protect cancer cells against chemotherapy and environmental stress.^88^ In addition, autophagy induced by lncRNAs has a key role in the proliferation and survival of tumor cells mediated by CAFs.^94^

##### Exosomal lncRNAs and metabolic programming

Metabolic activity in cancer cells is different from healthy cells.^88^ In hypoxia conditions, healthy cells begin to perform aerobic glycolysis (Warburg effect), while cancer cells are highly dependent on it.^95^ The cancer cells which are proliferated continuously require rapid production of ATP.^88^ Furthermore, the rapid production of ATP leads to the synthesis of many glycolytic intermediates which go to subsidiary pathways and supply energy for proliferating cancer cells.^96^ In the metabolic reprogramming of cancer cells, several signaling pathways play a role, including, phosphatidylinositol 3‐kinase (PI3K)/Akt, c‐Jun N‐terminal kinase (JNK), extracellular signal‐regulated kinase (ERK), and Ras.^97^ Because lncRNAs can regulate these signaling pathways, lncRNAs can affect the metabolism of cancer cells.^88^

### 2.2 Extracellular matrix

The ECM is a complex and diverse network of more than a hundred proteins, including proteoglycans (heparan sulfate, chondroitin sulfate), fibrous proteins (elastin, collagen), glycoproteins (laminins, tenascin C [TNC], fibronectin 1 [FN1]), and glycosaminoglycans (hyaluronic acid), which plays a role as a scaffold of organs and tissues, as well as constitutes the largest component of the TME.^6^, ^19^, ^98^ Although many tumor cells and diverse types of stromal cells can produce ECM proteins, CAFs have a main and significant role in the organization and composition of ECM.^99^, ^100^ Moreover, the ECM contains cytokines, chemokines, and growth factors released by stromal and tumor cells.^6^ The ECM regulates cell behavior and plays an important role in tissue function and maintenance.^101^, ^102^ Accordingly, disruption of the mechanisms involved in the regulation of ECM degradation, production, and remodeling causes pathological conditions such as cancer and fibrosis.^101^, ^103^, ^104^ Furthermore, necessary signals for cellular differentiation, growth, and migration are provided by ECM.^102^ Each component of the ECM, through cell surface receptors, induces signaling pathways to cells and plays a role in tumor events including differentiation, survival, migration, and metabolism.^105^, ^106^ Moreover, ECM heterogeneity provides evading growth suppressors, resisting cell death, and sustaining growth signals for cells, as a result, has a key role in tumor proliferation, also in tumor angiogenesis, metastasis, and invasion.^106^, ^107^ The ECM is responsible for cellular migration out of the TME and cellular adhesion, although it functions as a physical scaffold for cells.^19^ The presence of stored diverse soluble factors such as growth factors, cytokines, chemokines, and angiogenic factors in the ECM creates a continuous inflammatory condition that ultimately leads to the expansion of the cellular repertoire.^19^, ^108^, ^109^ In this inflammatory condition, the deposition of a large amount of ECM protein occurs via facilitation in the transformation of stromal fibroblasts into myofibroblasts, which results in contraction and an increase in stiffness.^109^, ^110^ One of the determining and important factors in TME involved in cancer treatment resistance is cell adhesion to ECM.^111^ The interactions of ECM components such as laminin, fibronectin, and collagen with integrin provide a context for drug resistance mediated by cell adhesion.^111^ The efficacy of many drugs depends on the composition of the cancer's ECM.^102^ The dynamic adaptation of ECM is involved in the invasion and progression of cancer and especially drug resistance, for instance, ECM can adequately hinder drug delivery by increasing the remodeling of microvascular ECs.^112^ Indeed, ECM remodeling creates a physical obstacle that delays or prevents drug delivery, thereby it increases drug resistance.^113^ Furthermore, ECM can activate survival proteins through survival pathways including MAPK, p53, PI3K/AKT, and subsequently promote chemoresistance.^102^ Alterations in stiffness and elasticity of ECM impact drug delivery to cancer cells, as well as pressure and diffusion, are factors related to drug delivery in the interstitial spaces.^102^ Drugs are delivered to tumors through the pressure of blood circulation and the interstitial areas.^111^ In the interstitial areas, the ECM organization causes an increase of fluid pressure through the physical obstacles of the tumor mass and subsequently extremely prevents the desired results of drug delivery.^111^ In addition, an increase in the fluid flux from the neoplasms to the surrounding environment due to the ample proliferation of cancer cells prevents the adequate transfer of drugs.^114^ Indeed, the density of ECM cells plays a key role in the low efficiency of drug delivery.^115^

Although all ECM components play a key role in tumor progression, the role of collagen stands out.^98^ Collagen can affect the behavior of tumor cells through discoidin domain receptors, tyrosine kinase receptors, integrins, and several signaling pathways.^116^ Furthermore, collagen can affect the activity of cancer cells by interacting with ECM molecules including MMPs, lamins, fibronectin, and hyaluronic acid.^117^ Exosomes and miRNAs are other components that have close interactions with collagen in cancer.^118^, ^119^ In the status that tumors are rich in collagen, the hypoxic condition is common, leading to the promotion of cancer progression.^116^ However, heparan sulfate (CGKRK peptide), gelatin (anginex, small geletic‐1 binding peptide), laminin (IKVAV), fibronectin extra domain A and B (anti‐EDB aptide), and aggrecan (a conjugate of quaternary ammonium) are some components of ECM that have therapeutic value.^98^

### 2.3 Cancer‐associated fibroblasts

Non‐malignant stromal cells in the TME are crucial for drug resistance and tumor progression.^112^ Stromal cells are polarized by their interaction with tumor cells, and through education of tumor cells, they secrete diverse molecules, resulting in pharmacokinetics regulation, immunosuppression, and metabolic regulation, subsequently, they reduce therapeutic outcomes.^112^ The most common type of stromal cells are CAFs. CAFs by involvement in ECM remodeling and metabolic reprogramming, as well as by secretion of diverse factors such as cytokines (Interleukin‐6 [IL‐6], IL‐8, IL‐10, etc.), growth factors (hepatocyte growth factor, stromal cell‐derived factor, fibroblast growth factor, transforming growth factor), various chemokines, metabolites, and exosomes (contain lncRNAs, miRNAs and mitochondrial DNA [mtDNA]) which activate multiple signaling cascades, cause drug resistance, and ultimately tumor recurrence^111^, ^112^, ^120^ Figure 2.

### 2.4 Mesenchymal stem cells

Mesenchymal stem/stromal cells have the potential for self‐renewal and differentiation into various cell types.^102^, ^106^, ^112^ Moreover, MSCs can be involved in many cancer events such as EMT, angiogenesis, anti‐apoptosis, metastasis, immunosuppression, pro‐survival, and treatment resistance especially drug resistance.^112^ These cells can be inherently resistant to chemotherapy and in various cancer types, they expand this resistance among cancer cells.^121^, ^122^, ^123^, ^124^, ^125^, ^126^, ^127^ MSCs can contribute to drug resistance and quiescence of cancer cells by creating a pre‐metastatic niche for tumor cells.^128^ Furthermore, they can move to inflammatory areas, and subsequently, infiltrate the tumor.^106^ Indeed, MSCs migrate to the tumor site via the action of factors derived from tumor cells, then, infiltrate the tumor and produce the necessary factors for cancer cells.^102^ MSCs can cause drug resistance through the following actions:

1. Direct cell‐to‐cell contact between MSCs and cancer cells that triggers multiple signaling cascades in tumors.^112^, ^129^
2. Genetic mutations in MSCs: Genetic changes occur not only in tumor cells but also in non‐malignant cells, leading to latent tumor recurrence in radiotherapy‐ and chemotherapy‐treated patients.^112^, ^130^
3. Secretion of soluble factors: MSCs can release a variety of fatty acids, cytokines, and growth factors that result in drug resistance.^102^, ^112^, ^131^, ^132^
4. Differentiation of MSCs into CSC or CAF: Some characteristics of CSCs are enhancing colony‐forming capacity and pluripotency, obtaining drug resistance along with loss of anchorage dependence, having the capability to promote of metastasis and tumor progression, and inherently resistant to chemotherapy, moreover, CAFs have a key role in drug resistance and CAF‐MSC cells (a type of CAF cells possessing resembling function and phenotype of bone marrow‐derived mesenchymal stem cell [BM‐MSC] in the tumor stroma) are involved in tumor growth, the decline in cell apoptosis, increase in cell proliferation, and resistance to chemotherapy; therefore, by differentiating MSCs into CSC or CAF, they can cause treatment resistance.^112^
5. Release of exosomes: According to multiple studies, exosomes released from MSCs promote chemotherapy resistance through mediation in interactions between cancer cells and MSCs, specific mRNA molecules and proteins transportation, and drug sequestration.^112^, ^133^

### 2.5 Cancer‐associated adipocytes

Cancer‐associated adipocytes (CAAs) are a type of stromal cells and an important factor in TME that play a role in resistance to apoptosis, angiogenesis, metastasis, drug resistance, and cancer cell invasion.^134^, ^135^, ^136^ CAAs promote cancer cell malignancy and tumor growth by secreting pro‐inflammatory cytokines, hormones, adipokines, adiponectin, resistin, and leptin.^109^, ^137^, ^138^ CAAs can also cause chemoresistance by secreting exosomes, for instance, exosomal miR21s derived from CAAs can inhibit apoptosis of cancer cells and promote chemoresistance by binding to apoptotic protease‐activating factor 1 (APAF1) in ovarian cancer.^62^, ^109^

In general, CAAs cause treatment resistance through the following actions:

1. Secretion of various factors: Adipose tissue acts both as a site for energy storage and as an endocrine organ, producing a wide range of exosomes, adipokines, leptin, growth factors, and adipocytokines that lead to treatment resistance.^112^, ^139^, ^140^
2. Extracellular matrix remodeling: CAAs are a crucial source for ECM components, also, the property of ECM dynamic adaptation has an important role in the invasion and progression of cancer, as well as drug resistance.^112^ CAAs release factors such as MMPs and collagen VI protein, resulting in the remodeling of ECM and chemotherapy resistance.^141^, ^142^, ^143^, ^144^ On the whole, according to several studies, lipid metabolism, and CAAs have an intricate role in the regulation of cancer sensitivity to anticancer drugs.^112^
3. Metabolism regulation: Since adipocytes perform energy storage, the metabolic relationship between tumor cells and adipocytes can naturally contribute to tumor progression.^112^ Similar to CAFs, the “Warburg effect” and “reverse Warburg effect” could also cause drug resistance mediated by adipocytes.^139^ The amount of lactate produced by adipocytes under hypoxia conditions increases significantly.^145^ Adipocytes also provide lipids for cancer cells to supply their main energy, which can cause treatment resistance and tumor progression, for instance, cancer cells take up exogenous free fatty acids released by adipocytes and these endogenous lipid molecules enhance the rate of fatty acid β‐oxidation (FAO), leading to extensive synthesis of ATP.^112^, ^146^
4. Alteration in the pharmacokinetics of chemotherapy: Pharmacokinetics of chemotherapy is altered by CAAs commonly via two actions: (a) Increase in drug clearance, (b) Alteration in drug distribution.^112^

Since the concentration of active drugs during treatment plays a key role in the efficacy of cancer therapy, in adipocyte‐rich microenvironments such as adipose tissue, it is observed a local decrease in cytotoxic chemotherapy activity due to decreased concentration, which could contribute to chemoresistance.^112^, ^147^

### 2.6 Acidosis

Acidification of the TME is related to metabolic adaptation and reprogramming.^148^ Moreover, tumor acidosis affects metastasis, invasion, and therapeutic response and can act as a regulator of tumor progression.^148^ Tumor cells have high metabolic demands that lead to the accumulation of a high amount of n the TME, and due to the disordered nature of tumor vasculature, effective removal of $H^{+}$ from the extracellular environment is prevented, accordingly, the accumulation of $H^{+}$ ions in the TME is intensified.^148^, ^149^ Consequently, it causes hypoxic conditions and changes in glycolytic metabolism. In addition, in areas where oxidative conditions prevail, the accumulation of $H^{+}$ causes the hydration of carbon dioxide, which meets the bioenergetic and biosynthetic needs of cancer cells, these processes happen at a high speed.^148^ Low pH in TME can promote the motility of cancer cells and subsequently, influence fibroblasts and activity and polarization of macrophages by bringing about alterations in cytoskeletal dynamics.^150^ In tumor regions where the lowest pH conditions prevail, the highest rate of invasion can occur and vice versa.^148^ To adapt to the acidic environment, tumor cells employ all enzyme systems.^106^ In general, the establishment of acidic conditions in the TME involves two events: CO_2_ by respiration and lactic acid formed by glycolytic metabolism.^106^ Evidence shows that acidic conditions overlap both in hypoxic areas and at the tumor‐stroma interface, which strongly influences tumor invasion and proliferation.^151^ Moreover, according to multiple evidence, the conditions of extracellular acidosis create a suitable and beneficial environment for dormant tumor cells to support the survival of disseminated tumor cells and the formation of metastasis, and subsequently, the chemotherapy‐resistant phenotype is maintained.^106^, ^152^

### 2.7 Metabolic reprogramming

Changes in energy metabolism are one of the specifications of tumor cells since tumor cells proliferate abnormally and accordingly, are dependent on the increased adaptation to the nutritional microenvironment, which is operated by the metabolic reprogramming.^112^, ^153^ Due to the glucose deficiency in the TME, the metabolism of cancer cells shifts to aerobic glycolysis (the Warburg effect).^154^ The final product of this metabolic process is lactate, which is associated with treatment resistance.^112^, ^155^ According to some evidence, other metabolic features such as reversed Warburg effect, glutamine metabolism, and metabolic symbiosis can cause acquired or adaptive drug resistance and challenge anti‐tumor treatment.^156^

Evidence suggests that disordered metabolism is involved in metastasis and malignancy.^111^ Severe reduction of amino acids such as glutamine, tryptophan, and arginine and high levels of glycolysis are observed in cancer cells.^111^ The metabolic features of malignant cells are regulated by tumor metabolic stress, leading to acidity, nutrient deficiency, and oxygen competition in the TME.^157^ Apart from epigenetic and genetic alterations of cancer cells, the interactions between various components of TME and metabolic competition contribute to drug resistance and the growth and metastasis of tumor cells.^158^, ^159^

### 2.8 Interstitial fluid pressure

Abnormal lymphatic and blood vessels cause acidic pH, hypoxia, and high interstitial fluid pressure (IFP) in TME and elevated IFP is a key player in preventing adequate drug delivery to solid tumors.^106^ Factors such as abnormal ECM, high cell density, disruption of lymphatic or venous drainage, and enhanced vascular permeability cause high IFP in the tumor.^160^ Abnormal proliferation of cancer cells leads to mechanical compression of blood vessels and lymphatic vessels in the confined area of the microenvironment, subsequently, meager lymphatic drainage and poor blood flow are caused, leading to abnormal vascular structures and a further reduction in a number of functional lymphatic vessels.^161^ As a result of excess fluid leakage from the vascular system into interstitial space or interstitium (where accumulates and swells the elastic ECM), IFP is increased, which is higher than in normal tissue.^106^

The amount of drug entering the tumor and its absorption by cancer cells affects the efficacy of chemotherapy.^162^ For a drug to reach cancer cells through blood vessels, it must be transferred from blood vessels to interstitial fluid and then from interstitial space to tissues.^163^ Therefore, the amount of IFP is important for drug delivery, and high IFP conditions can reduce drug transfer.^162^ High IFP also compresses blood vessels, diverting blood away from the tumor center to the periphery, as a result, drug delivery is further reduced.^162^

## 3 IMMUNE CELLS

The immune system plays a crucial role in monitoring cancer and responding to chemotherapy. MDSCs, TAMs, dendritic cells (DCs), regulatory and effector T cells, B cells, and natural killer (NKs) cells are the main immune cells present in the TME.^15^, ^164^ These cells can have either opposing or stimulating effects on the tumor and play a key role in tumorigenesis.^15^ Immune cells also contribute to the development of chemoresistance.^165^ According to several studies, the relationship between MDSCs and malignant cells has a significant impact on chemotherapy resistance and immune system suppression.^112^, ^166^, ^167^ At different stages of tumor development, diverse populations of T cells are observed in the TME.^168^, ^169^ An increase in T‐regulatory lymphocytes (T‐reg) infiltration in the TME is associated with chemoresistance in several types of cancer, including colorectal, lung, kidney, head and neck squamous cell carcinoma (HNSCC), melanoma, ovarian, and glioblastoma cancer.^170^, ^171^, ^172^, ^173^, ^174^, ^175^, ^176^ Evidence suggests that tumor cells may manipulate local DCs to form suppressive T cells, ultimately leading to drug resistance.^177^, ^178^ Drug resistance and tumor progression can be caused by high infiltration of tumor‐associated neutrophils (TANs) in the TME.^179^ TANs can lead to acquired drug resistance in cancer due to their capacity to increase angiogenesis, increase tumor cell proliferation, and suppress the immune system.^180^, ^181^ Moreover, TANs can reduce the efficacy of many cancer drugs such as common cytotoxic drugs and immune checkpoint inhibitors by activating various signaling pathways.^168^ Although NK cells may exhibit multi‐drug resistance‐like activity, according to studies, this property can be inhibited by drugs such as solutol HS‐15 or verapamil.^168^, ^182^, ^183^, ^184^

TAMs are derived from circulating monocytes and are one of the most abundant cells in solid tumors that have a significant impact on suppressing the immune system in the TME, furthermore, TAMs play a role in chemoresistance and tumor development.^111^ Among the immune cells present in the TME, macrophages have a prominent and critical role in chemoresistance due to their capabilities and high numbers. TAMs are the most abundant among immune cells in the TME, and their increased infiltration into the microenvironment leads to unfavorable outcomes in chemotherapy.^112^, ^185^, ^186^ Generally, TAMs are divided into two subgroups, M1 and M2, with the M2 phenotype playing a role in promoting drug resistance and tumor progression.^112^, ^187^ In cancer conditions, macrophages are educated by the TME, and TAMs are usually polarized from M1 to M2.^188^ Due to the interaction between TAMs and tumor cells, under the pressure of treatment, TAMs are promoted by tumor cells and differentiate into immunosuppressive M2‐polarized macrophages, leading to therapeutic resistance.^112^ Moreover, studies have demonstrated the type of M2 can lead to acquired drug resistance in cancer cells.^189^, ^190^, ^191^

TAMs, like CAFs, release a wide range of soluble factors in the TME, including chemokines, enzymes, interleukins, exosomes, and so forth, to combat drug attacks, for instance, TAMs can prevent paclitaxel‐induced tumor cell death by expressing cathepsin S and B.^112^ Moreover, through overexpressed cytidine deaminase or CDA (an enzyme that is involved in gemcitabine degradation), TAMs can promote chemotherapy resistance in cancer.^165^, ^192^ By secreting exosomal miR‐365, TAMs can increase the metabolism of gemcitabine in cancer cells, which ultimately leads to apoptosis inhibition and tumor resistance promotion.^193^, ^194^ TAMs upregulate Gfi‐1 in tumor cells by TGF‐β secretion, which ultimately leads to reduced sensitivity of tumor cells to gemcitabine by inhibiting HMGB1 (high mobility group box 1) and CTGF (connective tissue growth factor) expression.^195^ By expressing IGF, TAMs can cause resistance to chemotherapy with albumin‐binding paclitaxel and gemcitabine.^165^, ^196^ Additionally, TAMs can cause chemotherapy resistance in pancreatic cancer by inducing EMT.^197^ According to evidence from prostate cancer treatment with ADT, CSCs can remodel macrophages into TAMs, subsequently, through the IL6/STAT3 signaling pathway, TAMs can increase stem‐like features of CSCs and drug resistance.^112^, ^198^

Overall, TAMs use various mechanisms to induce drug resistance, including regulating CSC properties, transforming into M2 suppressive phenotype, promoting EMT, releasing various cytokines, and suppressing immune cells.^112^

## 4 DNA REPAIR MECHANISMS

Preserving the genome and transmitting a healthy genome to the next generation is an essential task for living organisms.^199^ However, DNA is constantly exposed to both endogenous insults (such as intracellular free radical oxygen species [ROS], etc.) and exogenous genotoxic insults (such as ionizing radiation [IR], ultraviolet [UV] radiation, chemotherapeutic drugs, etc.), that can damage to DNA.^199^ Although DNA damage is a crucial target for radiotherapy and chemotherapy, it can also lead to the development of cancer.^199^ Therefore, to maintain genome integrity, living organisms rely on a complex system and multiple mechanisms to counteract DNA‐damaging factors, collectively known as the DNA damage response (DDR).^200^

Several DNA repair pathways are utilized to counteract DNA damage, including: (1) Base excision repair (BER), (2) nucleotide excision repair (NER), (3) homologous recombination (HR), (4) non‐homologous end joining (NHEJ), (5) mismatch repair (MMR), (6) microhomology‐mediated end joining (MMEJ), (7) DNA damage tolerance (DDT) (translesion synthesis [TLS], template switching [TS]), (8) $O^{6}$‐methylguanine‐DNA methyltransferase (MGMT) pathway, (9) Fanconi anemia (FA) pathway, (10) single‐strand annealing (SSA).^200^, ^201^, ^202^, ^203^

Among these pathways, BER, MMR, NER, HR, and NHEJ are the major and essential pathways in DNA repair.^200^, ^201^

Moreover, there are several types of DNA damage, including: (1) clustered damaged sites, (2) base damage, (3) single‐strand breaks (SSBs), (4) double‐strand breaks (DSBs), (5) sugar damage, (6) DNA cross‐linking.^204^

DSBs are the most destructive and the most deleterious type of DNA damage for cells which can bring about cell death or carcinogenesis.^204^ Among the types of DNA damage, SSBs and DSBs are prominent which can bring about genome rearrangement. The direct and indirect BER, MMR, and NER pathways repair SSBs damage, while the SSA, NHEJ, and HR pathways repair DSB damages.^200^, ^205^, ^206^ Moreover, DNA adducts and replication errors are repaired by the NER and MMR pathways, respectively.^200^

To control the DDR, cells utilize epigenetics and miRNAs as regulators. Epigenetic alterations in gene expression and tumor heterogeneity play a key role, as a result, epigenetic chromatin regulation can influence the mechanisms and pathways involved in the DNA repair process.^207^ Histone deacetylases (HDACs) can contribute to the preparation of chromatin for DSB repair promotion via NHEJ and HR.^207^ Furthermore, DNA methylation is a common epigenetic mechanism in cancer cells and gene inactivation.^207^ Alterations in gene promoter methylation status of DDR components are observed in diverse cancer types including oral squamous cell carcinoma, thyroid cancer, NSCLC, neck squamous cell carcinoma, gastric cancer, acute myeloid leukemia (AML), breast cancer, ovarian cancer, bladder cancer.^207^, ^208^, ^209^, ^210^, ^211^, ^212^, ^213^, ^214^, ^215^, ^216^, ^217^, ^218^ Moreover, the methylation status of some DDR genes can be employed as treatment response, prognostic, and diagnostic biomarkers in diverse types of cancer.^207^ miRNAs function as regulators in various processes, including tumorigenesis, and post‐transcriptional control of DNA repair components, in addition, miRNAs can regulate the expression levels of DNA repair genes and subsequently modulate the sensitivity of cancer cells to DNA‐damaging agents.^207^

**FIGURE 3:**
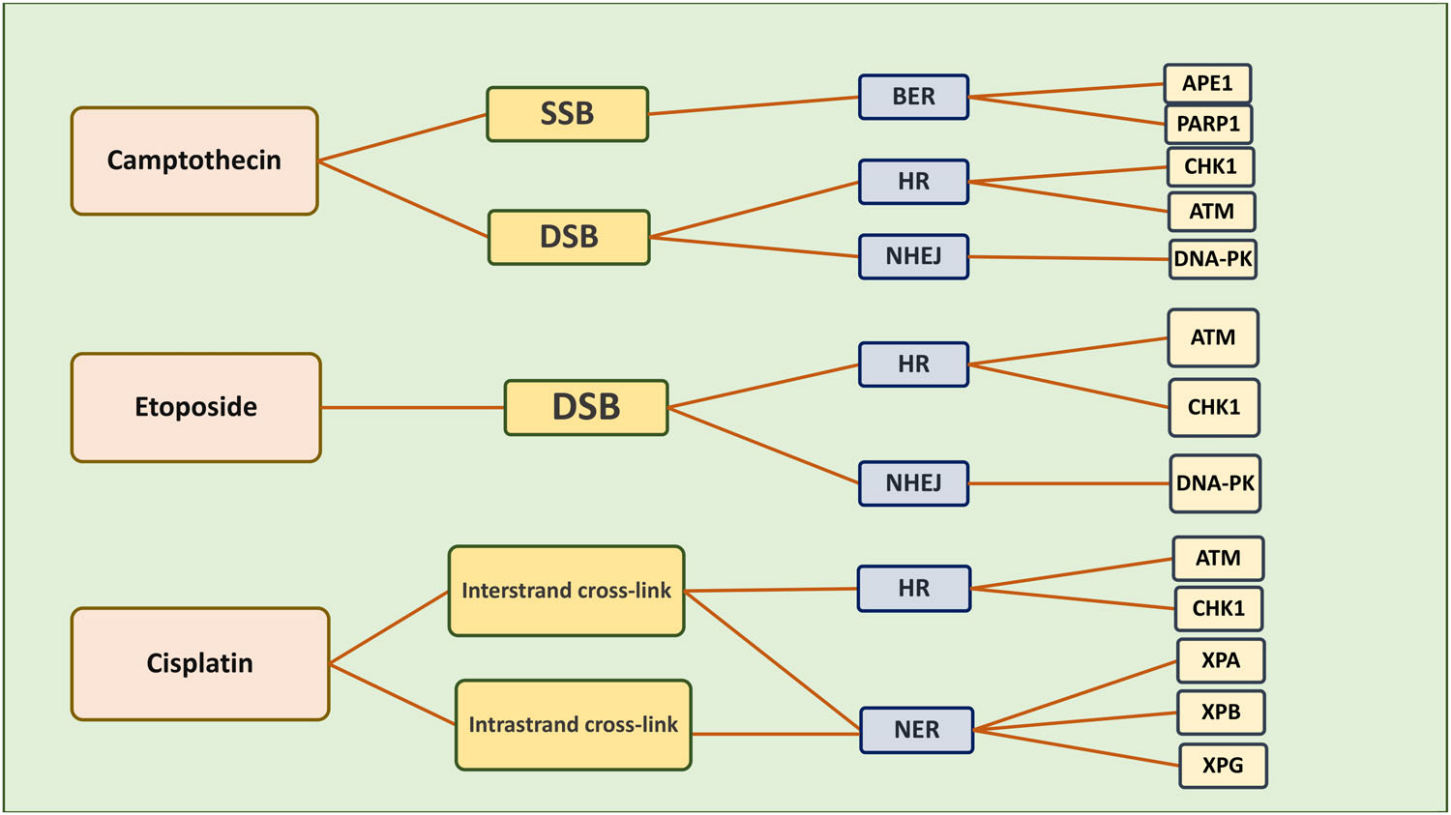
Performance of DNA repair mechanism pathways against camptothecin, etoposide, and cisplatin.

Chemotherapeutic agents commonly used include topoisomerase I inhibitors, alkylating agents (such as cisplatin), and DNA topoisomerase II inhibitors.^219^ During chemotherapy using camptothecin (a topoisomerase I inhibitor), if SSB damage occurs, the BER pathway is activated, and subsequently, PARP1 and APE1 enzymes are activated, however, if DSB damage occurs, the HR and NHEJ pathways are activated, followed by the HR pathway activating ATM and CHK1 enzymes, and NHEJ activating DNA‐PK enzyme.^202^ When etoposide (a topoisomerase II inhibitor) is prescribed, DSB damage occurs, which activates the HR and NHEJ pathways.^202^ The HR pathway activates ATM and CHK1 proteins, and NHEJ activates DNA‐PK.^202^ When Cisplatin (an Alkylating agent) is prescribed, DNA interstrand cross‐link (ICL) damage (activating HR and NER pathways) and intrastrand cross‐link damage (activating NER pathway) occur.^202^ Then, the HR pathway activates ATM and CHK1 proteins, and the NER pathway activates XPA, XPB, and XPG proteins^202^ Figure 3.

## 5 CANCER STEM CELLS

One of the reasons for cancer progression and treatment failure is cancer heterogeneity.^220^ Different cancer cell types in the tumor contribute to tumor heterogeneity, and among these cells, CSCs are highly involved in the initiation and progression of cancer as well as have self‐renewal and differentiation abilities.^221^ Stem cells in cancers can be divided into two categories based on their function:

1. Resident CSCs that can initiate the tumor;
2. Migratory stem cells that metastasize and form tumors in another location.^221^

Due to these capabilities, CSCs play a key role in tumor initiation, drug resistance, metastasis, and cancer recurrence.^222^ During successful chemotherapy, although a significant portion of tumor cells undergo apoptosis, a subset of CSCs may survive and cause cancer recurrence.^223^, ^224^

There is a wide range of mechanisms and factors that contribute to CSCs to promote chemoresistance, including:

1. Tumor microenvironment (autophagy, inflammation, adipocyte‐released factors, CAFs, MSCs, ECM, hypoxia, ECs, immune cells);
2. EMT induction or activation of EMT‐transcription factors;
3. Self‐renewal ability (high telomerase activity);
4. High expression of CSCs markers (such as CD133, ALDH1, CD44, CD24+);
5. Quiescence/dormancy/low proliferation rate;
6. Stemness genes (such as Bmi1 and Musashi [MSI]);
7. Epigenetic mechanisms (DNA methylation, histone modifications);
8. Signaling pathways (such as Hedgehog pathways, Notch pathways, and Wnt pathways);
9. Resistant to DNA damage‐induced cell death (promoting the DNA repair capability, enhancing ROS scavenging, activating the anti‐apoptotic signaling pathways);
10. Metabolism alteration;
11. Higher expression of multi‐drug resistance (MDR) or detoxification proteins (aldehyde dehydrogenase [ALDH], drug‐transporter proteins [ABCG1, ABCB1]);
12. Non‐coding RNAs (ncRNAs).^220^, ^225^, ^226^, ^227^, ^228^, ^229^, ^230^

There is extensive evidence indicating that ncRNAs, including lncRNAs and miRNAs, play a key role in regulating CSC capabilities such as asymmetric cell division, cancer recurrence, tumor initiation, self‐renewal, and drug resistance.^220^, ^231^, ^232^, ^233^, ^234^, ^235^, ^236^ Moreover, according to multiple studies, ncRNAs control the cancer progression and growth and division of CSCs by regulating downstream signaling pathways and transcription factors.^226^, ^237^, ^238^, ^239^, ^240^

## 6 DISCUSSION AND CONCLUSION

Cancer cells employ a wide range of factors and mechanisms to resist chemotherapy. More important than the wide number of factors and components involved in therapeutic resistance is the extensive cooperation and communication between various components of the TME and tumor cells, which play a key role in making cancer treatment challenging, for example, when a group of cancer cells becomes sensitive to treatment, this cooperation between different factors that cause the reduction of this sensitivity and finally the failure of the treatment. These components, with the help and strengthening of each other, make a network of resistance to treatment in such a way that it is very complicated and challenging to break it. For this reason, the idea of combined treatments has been considered to break this resistance network by using different treatment approaches and expanding the number of factors that are targeted. However, this idea is still far from the desired results. According to the evidence presented in this article, through shifting cancer research to better understanding interactions and communications of factors involved in therapeutic resistance in order to enhance the knowledge about factors such as ncRNAs that play an important role in intracellular and extracellular processes, potential targets can be achieved such that by targeting them, the resistance network would be broken and favorable results would be seen in cancer treatment. In other words, it is likely that by targeting components that play a significant role in the communication and cooperation of factors involved in treatment resistance, components of the TME and cancer cells become highly vulnerable to treatment, furthermore, CSCs can no longer play their well‐known prominent role in treatment resistance by influencing other factors.

Among the factors that play a role in chemotherapy resistance, the triad of CSCs, exosomes, and ncRNAs are particularly prominent. CSCs not only utilize diverse intracellular and extracellular mechanisms to develop chemoresistance but also have a significant role in chemoresistance through their interactions with various components of TME and other cancer cells. Furthermore, It has also been established that CSCs play a crucial role in recurrence after successful chemotherapy. As previously mentioned, exosomes present in the microenvironment have a prominent role in cellular communications and interactions between different components of TME and cancer cells. Additionally, ncRNAs have an important role in intracellular signaling pathways and intercellular communications between CSCs and other factors. Therefore, it can be deduced that ncRNAs are likely to have a significant impact on the function of mentioned factors involved in chemotherapy resistance caused by CSCs. Moreover, CSCs can utilize their high capacity of survival and chemoresistance to stay alive after chemotherapy, as well as employ the combination of exosomes and ncRNAs to spread chemoresistance among other cancerous cells by establishing wide intercellular communications with tumor cells and the TME components, ultimately leading to the exacerbation of chemotherapy resistance and cancer relapse. Indeed, ncRNA can be considered the most prominent factor among other factors in the chemoresistance of CSCs. Therefore, an in‐depth understanding of the interactions between CSCs, exosomes, and ncRNAs is very important and vital in achieving the desired chemotherapy results, and research in these three fields is very necessary to understand their precise function in chemotherapy resistance. According to Figure 1, none of the approved small molecule drugs target ncRNAs which are very important factors in intracellular and extracellular signaling in cancer rather scientists have focused on other targets for designing these drugs. Until now, the therapeutic results of these drugs have not been as satisfactory as expected from targeted therapy. Given the research and evidence reviewed in this article, by targeting exosomal ncRNAs and CSCs with targeted therapies such as small molecules, the resistance network that exists in cancer can collapse or be weakened so much that the outcomes of combination therapies would become improved so that instead of the increasing rate of death from cancer, this trend would decrease in the future.

In addition, it is recommended that the use of smart nanoparticles as a drug delivery method for small molecules could be an effective approach for delivering a greater and more fruitful amount of small molecules to cancer cells, considering the distinct conditions within the TME (hypoxia, acidity, etc.). One of the challenges in drug delivery is the limited understanding of drug targets. Large‐scale screening studies using CRISPR‐Cas technology can help to improve our understanding of high‐priority drug targets.

On the whole, the application of the following strategies can improve the design and therapeutic outcomes of small molecule drugs as combination therapy for chemotherapy:

1. Focusing more research on interactions between CSCs, exosomes, and ncRNAs as well as their function in chemotherapy resistance for a better and deeper understanding of these areas.
2. Identifying the factors that maintain proper function and favorable interactions between CSCs, exosomes, and ncRNAs to be considered as targets for small molecule drugs so that targeting them causes effective disruption in the function and interactions of these three components.
3. Launching extensive screening studies using CRISPR‐Cas technology to identify potential drug targets for the design of effective small molecule drugs.
4. More research on drug delivery systems based on smart nanoparticles to improve and increase their efficiency in the appropriate and sufficient delivery of small molecule drugs to the TME and cancer cells.

There may be some possible limitations in this review article. The aim of this article is to highlight the role of ncRNAs, exosomes, and CSCs in chemotherapy resistance, in addition to introducing them as therapeutic targets. Although it has been tried to review the factors involved in chemotherapy resistance via citing 240 references, these are the main factors in chemotherapy resistance, not all. Furthermore, since the aim of this article is to present a new approach to designing targeted treatments, especially small molecule drugs against CSCs and targets that play a crucial role in intercellular communication in cancer, all the information related to the factors involved in chemotherapy resistance is not mentioned. In other words, a separate article can be written about each of these factors, but this article reviews studies in such a way that scientists who are in the field of targeted therapies, especially designers of small molecule drugs pay special attention to the role of intercellular communication and CSCs as targets in cancer treatment.

## AUTHOR CONTRIBUTIONS

**Amirhossein Rismanbaf:** Investigation (equal); project administration (equal); writing – review and editing (equal).

## CONFLICT OF INTEREST STATEMENT

The authors have stated explicitly that there are no conflicts of interest in connection with this article.

## ETHICS STATEMENT

None.

## Data Availability

The data that support the findings of this study are available on google scholar.
